# Correlation between Oral Glucose Tolerance Test Abnormalities and Adverse Pregnancy Outcomes in Gestational Diabetes: A Cross-Sectional Study

**DOI:** 10.3390/ijerph17196990

**Published:** 2020-09-24

**Authors:** Aida Kalok, Ming Yean Ong, Aqilah Hasrori, Ker Shing Chiang, Fatin Yazim, Salahuddin Baharuddin, Rahana Abdul Rahman, Shamsul Azhar Shah, Nor Haslinda Abd Aziz, Shuhaila Ahmad, Nor Azlin Mohamed Ismail

**Affiliations:** 1 Department of Obstetrics and Gynaecology, Faculty of Medicine, National University of Malaysia, Universiti Kebangsaan Malaysia Medical Center, Cheras 56000, Kuala Lumpur, Malaysia; drrahana@ppukm.ukm.edu.my (R.A.R.); norhaslinda.abdaziz@ppukm.ukm.edu.my (N.H.A.A.); shuhaila@ppukm.ukm.edu.my (S.A.); azlinm@ppukm.ukm.edu.my (N.A.M.I.); 2 Faculty of Medicine, National University of Malaysia, Universiti Kebangsaan Malaysia Medical Center, Cheras 56000, Kuala Lumpur, Malaysia; mingyean96@gmail.com (M.Y.O.); aqilahhasrori@gmail.com (A.H.); kershing0814@outlook.com (K.S.C.); fatin_yazim@yahoo.com (F.Y.); salahuddinbaharuddin@gmail.com (S.B.); 3 Department of Community Health, Faculty of Medicine, National University of Malaysia, Universiti Kebangsaan Malaysia Medical Center, Cheras 56000, Kuala Lumpur, Malaysia; drsham@ppukm.ukm.edu.my

**Keywords:** glucose tolerance test, gestational diabetes, pregnancy outcome

## Abstract

Gestational diabetes mellitus (GDM) is associated with maternal and neonatal complications. We aimed to evaluate the relationship between the abnormalities of the oral glucose tolerance test (OGTT) and adverse pregnancy outcomes. This was a retrospective study of GDM patients over a five-year period in a Malaysian tertiary center. The diagnosis of GDM was based on the National Institute for Health and Care Excellence (NICE) guideline. The data on patients’ demographics, OGTT results, GDM treatment, and pregnancy outcomes were analyzed. A total of 1105 women were included in the final analysis. The percentage of women with isolated abnormal fasting glucose, isolated two-hour abnormality, and both abnormal values were 4.8%, 87.1%, and 8.1%, respectively. Women with both OGTT abnormalities had a higher risk of preeclampsia (odds ratio (OR) 4.73; 95% confidence interval (CI) 1.45–15.41) and neonatal hypoglycemia (OR 8.78; 95% CI 1.93–39.88). Isolated postprandial abnormality was associated with an 80% lesser risk of neonatal hypoglycemia (OR 0.19; 95% CI 0.04–0.87). Both isolated fasting and multiple OGTT abnormalities were associated with insulin therapy. Multiple OGTT abnormalities were a positive predictor of adverse pregnancy outcomes, while isolated postprandial abnormality was associated with a lesser risk of neonatal complication. Further prospective study is essential to validate these findings.

## 1. Introduction

Gestational diabetes mellitus (GDM) is defined as diabetes diagnosed in the second or third trimester of pregnancy that was not clearly overt diabetes prior to gestation [1]. GDM is associated with significant maternal and fetal morbidities. Mothers with GDM are at increased risks of preeclampsia, polyhydramnios, preterm birth, and cesarean delivery, while fetal and neonatal complications include macrosomia, shoulder dystocia, neonatal jaundice, and hypoglycemia [2]. Studies have shown that rising maternal glucose leads to a graded increase in the risk of complications, even within the accepted normal plasma glucose range [3,4]. Treatment of GDM has been proven effective in decreasing or preventing maternal and fetal short-term complications, in particular, preeclampsia and fetal macrosomia [5,6].

The increasing prevalence of obesity, sedentary lifestyle, and unhealthy diet contribute to the global rise in GDM [7]. The documented prevalence of GDM worldwide varied substantially from 1% to more than 30%. The lack of universally accepted screening and diagnostic criteria for GDM has made international comparisons difficult [2]. Various international bodies, including the World Health Organization (WHO), advocate screening all pregnant women for diabetes, while the British National Institute for Health and Care Excellence (NICE) supports risk-based screening [8,9]. The diagnostic thresholds for GDM also vary between countries. The International Association of Diabetes in Pregnancy Study Groups (IADPSG) criteria for the diagnosis of GDM have been recognized by the WHO, and recently by the International Federation of Gynecology and Obstetrics (FIGO) [10,11]. Institutions, such as NICE and the American College of Obstetrics and Gynecology, however, have not endorsed these criteria.

Although fasting plasma glucose values demonstrated a stronger link to poor pregnancy outcomes than the 1 h and 2 h values [2], the exact relationship between different characteristics of the oral glucose tolerance test (OGTT) and pregnancy outcomes remain unclear [12]. The degree of association between maternal glycemia and pregnancy outcomes in different populations could vary, for example, by ethnicity [11]. A study from China demonstrated a clear association between fasting hyperglycemia and fetal macrosomia as well as cesarean delivery, while the two-hour OGTT abnormality was significantly linked to preterm birth [12]. The data on the population from South East Asia are currently limited to research from Australia as well as the landmark Hyperglycemia and Adverse Pregnancy Outcome (HAPO) study [13,14]. The objective of our study was to assess the relationship between the OGTT abnormalities and pregnancy outcomes among Malaysian urban pregnant women. Understanding the correlation between the OGTT parameters and specific adverse pregnancy outcomes is essential in planning blood glucose monitoring and early commencement of treatment with the aim to reduce overall complications rate.

## 2. Materials and Methods

This was a retrospective study of pregnant women with GDM who delivered in a tertiary center in Kuala Lumpur, Malaysia, over a five-year period from January 2014 to December 2018. Ethical approval was obtained from the UKM Medical Research and Ethics Committee (Research Code: JEP-2019–268) before data collection. The diagnosis of GDM was based on the NICE guideline (fasting glucose ≥ 5.6 mmol/L and/or two-hour post glucose load ≥ 7.8 mmol/L) at any gestation with one-step 75 g OGTT. Women with multiple pregnancy and overt diabetes (fasting glucose > 7.0 mmol/L, two-hour glucose > 11.1 mmol/L) were excluded. The GDM women were identified from our institution’s birth registry. The demographics and clinical data were obtained from multiple sources, i.e., medical records, electronic labor, and discharge summaries, as well as an electronic reporting system. Cases with incomplete data were excluded from the analysis. Women’s demographics, maternal risk factors, OGTT results, GDM treatment, mode of delivery, and pregnancy outcomes were recorded. The main outcome measures were cesarean delivery, preeclampsia, defined as any hypertension with proteinuria, which developed after 20 weeks of gestation. Preterm birth was defined as any delivery before 37 completed weeks of gestation, while stillbirth was defined as in utero fetal death after 24 weeks of gestation. Neonatal complications, such as macrosomia (defined as a birth weight of more than 4000 g), neonatal hypoglycemia, neonatal jaundice, and admission to the Neonatal Intensive Care Unit (NICU) were also recorded.

### Statistical Analysis

The study data were analyzed using the IBM SPSS Statistics for Windows, version 24 (IBM Corp., Armonk, NY, USA). Data were presented as mean (standard deviation, SD) or percentage for continuous and categorical variables, respectively. Categorical variables were analyzed using the chi-square test (or Fisher’s exact test if the expected value was less than 5), whereas an independent *t*-test was performed to compare numerical data. The one-way ANOVA with Tukey honestly significant difference adjustment for multiple comparisons, was used to compare means of more than two independent groups. The binary logistic regression model was used to analyze the odds ratios (OR) of each OGTT abnormality in relation to GDM treatment, mode of delivery, and adverse pregnancy outcomes. Multiple logistic regression models were used to calculate adjusted odds ratios (AORs) and corresponding 95% CIs for the associations between each type of OGTT abnormality and all pregnancy outcomes, after controlling for maternal age, ethnicity, parity, and maternal obesity. A *p*-value < 0.05 was regarded as statistically significant. AORs were also calculated for the relationship between maternal risk factors and OGTT abnormalities.

## 3. Results

Our initial screening identified 1206 women. Nine cases were excluded due to incomplete data. Seventy-seven women were categorized as overt diabetes, and fifteen were diagnosed based on the IADPSG criteria (fasting between 5.1 to 5.5 mmol/L), making the number for final analysis 1105. Baseline demographics and risk factors are shown in Table 1. The mean maternal age was 32.2 years, while the mean gestation at diagnosis was 24.6 weeks, with a range from 9 to 36 weeks. The majority of women were diagnosed in the second trimester (53.7%), while around two-fifths of our cohort had positive OGTT in the third trimester. More than three-quarters of our GDM women were of Malay ethnicity. The proportion of women with abnormal fasting and two-hour post glucose load were 12.9% and 95.2%, respectively. The percentages of women with isolated abnormal fasting glucose, isolated two-hour abnormality, and both abnormal values were 4.8%, 87.1%, and 8.1%, respectively. The majority were managed by diet modification (87.6%), while the remaining required treatment by either metformin, insulin, or a combination of both.

There were significant differences among the different types of OGTT abnormalities in terms of mean fasting and postprandial glucose levels. Women with isolated two-hour abnormality had significantly lower mean fasting glucose in comparison to those with isolated fasting hyperglycemia (*p* < 0.001) and both abnormal OGTT values (*p* < 0.001). Interestingly, those with isolated fasting abnormality were found to have significantly lower mean postprandial glucose levels than the isolated two-hour (*p* < 0.001) and multiple OGTT abnormalities (*p* < 0.001). Insulin use was positively correlated with isolated fasting and combined abnormalities, even after adjustment for maternal age, ethnicity, parity, and obesity.

Table 2 demonstrates the relationship between the types of OGTT abnormality and pregnancy outcomes. There were no significant associations between isolated fasting hyperglycemia and any adverse pregnancy outcomes. The isolated two-hour post glucose load abnormality was associated with reduced risk of neonatal hypoglycemia (OR 0.19; 95% CI 0.04–0.87). The association, however, was not significant in the multivariable analysis. Combined OGTT abnormalities group demonstrated an increased risk of preeclampsia (OR 4.73; 95% CI 1.45–15.41) and neonatal hypoglycemia (OR 8.78; 95% CI 1.93–39.88). The positive correlations remained significant even after the adjustment for maternal age, ethnicity, parity, and obesity was made.

Around 8.7% of women in our cohort required insulin therapy. Table 3 depicts the clinical characteristics of insulin-treated women versus those on diet modification.

Women on insulin were diagnosed at earlier gestation (*p* < 0.001) and had significantly higher fasting blood glucose levels (*p* < 0.001). Maternal risk factors, such as obesity, previous GDM, and family history, were significantly more prevalent in the insulin-treated group. Insulin therapy was an independent predictor of cesarean delivery, preeclampsia, stillbirth, and neonatal hypoglycemia among our cohort.

Table 4 demonstrates the associations between GDM risk factors and OGTT abnormalities. Past GDM and maternal obesity were independent risk factors for multiple OGTT abnormalities, while family history was positively associated with an isolated postprandial abnormality. 

## 4. Discussion

Variation in the clinical characteristics of GDM had been observed in multi-ethnic studies. A retrospective cohort study in Australia found that South-East Asians had the lowest fasting glucose among five different ethnic groups. The prevalence of fasting abnormality from the multi-centered HAPO study was 55%. However, the participated institutions from the South-East Asia regions, such as Bangkok and Hong Kong, had a lower prevalence of 24% and 26%, respectively. Interestingly, the data from Hong Kong also showed the highest proportion of two-hour abnormal glucose levels on OGTT (65%) [13].

While data on the Caucasian population showed a low prevalence of abnormal two-hour OGTT values (7–9%) [13,15], several studies demonstrated marked postprandial hyperglycemia in East Asians compared with matched Caucasian subjects [14,16,17]. Our cohort demonstrated similar findings in which the majority of our women had abnormal two-hour abnormality with a relatively low proportion of fasting hyperglycemia. Although we did not use the IADPSG criteria for our cohort, the low prevalence of fasting abnormalities among our women was unlikely to affect the overall study findings. A previously published study from our institution found no significant difference in the prevalence of GDM between the women diagnosed using the 1999 WHO (fasting ≥6.1 mmol/L, 2-h ≥7.8 mmol/L) and IADPSG (fasting ≥5.1 mmol/L, 2-h ≥8.5 mmol/L) criteria [18], a result that could be attributed to the higher rate of postprandial abnormality in our population.

We found that fasting abnormality was a positive predictor for insulin therapy, a result similar to other studies [19,20,21,22,23,24,25]. The mean fasting glucose among our women on insulin was higher than those who were on diet therapy, with no significant difference noted in the postprandial level. Ares et al. found that the two-hour plasma glucose level was significantly higher in the insulin-treated group [20]. The study, alongside several others, discovered that the post-glucose load abnormalities were significant predictors of insulin requirement among GDM women [20,21,23,26,27]. We found that two-hour glucose derangement was significantly associated with diet therapy and reduced the risk of insulin usage. Our results were similar to that of the South East Asia ethnic group in the Australian population study, in which, despite having the highest two-hour glucose level, their insulin requirement was the lowest, and the majority of the women were managed by diet alone [14]. This suggests that most of our women with postprandial derangement were more likely to have mild gestational diabetes, which did not require escalation of treatment. Our study demonstrated that combined OGTT abnormalities were significantly associated with insulin therapy. Multiple abnormalities on OGTT were a positive predictor of antenatal insulin in GDM mothers [22,28,29,30,31]. Mitra et al. found that the prevalence of multiple OGTT abnormalities was four times higher in insulin-treated women compared to those in the non-insulin group (*p* < 0.001) [28].

The insulin requirement among our GDM women (8.7%) was low in comparison to other published data (10.8–52.8%) [5,20,21,22,26,27,28]. Singapore, which has a similar multi-ethnic population to Malaysia, reported a comparable rate to ours (5.6–10.4%) [32]. Studies have shown that the underlying disturbance in glucose metabolism was different between subjects with impaired fasting glucose (IFG) and impaired glucose tolerance (IGT) [33,34]. The Botnia study demonstrated that those with IFG were more insulin resistant, evidenced by significantly higher HOMA IR (Homeostatic Model Assessment of Insulin Resistance) and fasting insulin level. Elevated levels of triglyceride and total cholesterol in IFG were suggestive of a link to metabolic syndrome. IGT, characterized by an elevated two-hour glucose level on OGTT, was associated with impaired insulin secretion. Hanfield et al. found that IGT subjects exhibited a deficit in the early and late phases of insulin secretion [34]. Those with both abnormal values on OGTT were also found to demonstrate a higher degree of insulin resistance in comparison to subjects with isolated abnormalities [33]. The strong link between insulin requirement and fasting and multiple OGTT abnormalities could, therefore, be explained by the presence of insulin resistance. A study on GDM women by Benhalima et al. also showed similar findings in which the insulin-treated women (with higher fasting glucose) demonstrated significantly lower insulin sensitivity [22].

The landmark HAPO study found a significant association between fasting hyperglycemia and increased cesarean rate and large for gestational age (LGA) [35]. Multiple other studies have since demonstrated similar findings [12,29,36]. A systematic review by Farrar et al. showed that there were positive linear associations with cesarean section, induction of labor, large for gestational age, macrosomia, and shoulder dystocia for all glucose exposures in GDM women, with stronger associations seen in the fasting than in the post-load glucose concentration [11]. Unlike other studies, the lack of association between fasting hyperglycemia and adverse pregnancy outcomes in our cohort could be due to the small proportion of women with such abnormality.

Insulin treatment is associated with a higher rate of LGA [12,20,22,29]. The mean infant birthweight in our insulin group was non-significantly higher than that of diet. The small number of women on insulin or low level of insulin requirement among our cohort may provide an explanation for this. Association between multiple OGTT abnormalities and adverse pregnancy outcomes was also demonstrated by other studies [12,29,30]. Women with two or more elevated glucose values may have a more severe disruption in glucose metabolic balance and insulin sensitivity than those with a single hyperglycemic value on OGTT [29]. Unsurprisingly, our findings showed that multiple OGTT abnormalities were linked to a more severe form of GDM, which required insulin and positively associated with adverse pregnancy outcomes.

### Strengths and Limitations of This Study

To our knowledge, this study is the first to describe the relationship between OGTT abnormalities and adverse pregnancy outcomes for the Malaysian population. Although there are published data from counties such as Singapore, which has similar ethnicity to ours, the group distribution was different as three-quarters of our cohort were Malays, while the largest ethnic group in the Singaporean cohort was Chinese (56.7%) [37]. The ethnic Malay proportion in our cohort, however, was higher than that of the general population of Kuala Lumpur (41%, 37%, and 9% for Malay, Chinese, and Indian, respectively) [38]. The large sample size also contributes to the strength of this study.

Our research has a few limitations. The data collection was limited by its retrospective design. Information, such as socioeconomic status and booking body mass index (BMI) that may be useful to explain the nature of our findings, was lacking. Obesity (defined as BMI 27.5 kg/m^2^ or greater) was recorded as a risk factor in the electronic records, such as discharge summaries; however, the exact women’s BMI values were often absent as these were usually found in the women’s handheld antenatal books. The BMI threshold for obesity among Malaysians is lower than that defined by the WHO, as research has shown that the risk of comorbidities in the Asian population begins to increase at a lower BMI value [39].

HbA1c levels were not available for all women for analysis; this could be due to the large proportion of GDM women managed through diet modification. Women with overt diabetes were identified based on the OGTT results in the medical records and were excluded from our analysis. We could not exclude the possibility of undetected overt diabetes from our retrospective data.

Our five-year study period was from 2014 to 2018, during which two main guidelines for GDM were published, i.e., NICE and Malaysia Clinical Practice Guideline (CPG). The Malaysian CPG, published in 2015, adopted part of the IADPSG diagnostic criteria, with fasting glucose ≥ 5.1 mmol/L and 2-h plasma glucose ≥ 7.8 mmol/L. Our institution had used the NICE criteria for diagnosis. Fifteen women who fulfilled the IADPSG fasting criteria were excluded from our final analysis. These women were diagnosed as GDM elsewhere and received antenatal care at our institution. The small number of women with isolated fasting and multiple OGTT abnormalities in our cohort might have resulted in a non-significant association with adverse pregnancy outcomes. The wide range of gestation at which diagnosis of GDM among our subjects also affected the duration of treatment, hence the pregnancy outcome. Almost one-tenth of women on insulin were diagnosed in the first trimester, while around 70% had positive OGTT in the second trimester. We were unable to analyze the treatment duration due to the nature of our data collection. A future prospective multicenter study would be useful to determine the true prevalence of fasting abnormality among the Malaysian population as well as the effect of GDM treatment duration on pregnancy outcomes.

## 5. Conclusions

The majority of Malaysian GDM women exhibited postprandial hyperglycemia, which was sufficiently managed through diet modification. In keeping with previous studies, our study demonstrated that multiple OGTT abnormalities were positive predictors of insulin treatment and adverse pregnancy outcomes. Women with such abnormalities may benefit from close glucose monitoring and early initiation of treatment to reduce maternal and neonatal morbidities.

## Figures and Tables

**Table 1:** Clinical Characteristics for study population and subtypes of oral glucose tolerance test (OGTT) abnormalities.

Clinical Characteristics	Study Cohort (*n* = 1105)	Subtypes of OGTT Abnormalities			
		*i*-Fasting (*n* = 53)	*i*-2hour (*n* = 963)	Both Abnormal (*n* = 89)	*p* Value
Maternal Age (years), mean (SD)	32.2 (4.4)	31.1 (4.5)	32.1 (4.4)	33.2 (4.6)	0.02
Ethnicity, *n* (%) Malay	830 (75.1)	47 (90.4)	714 (74.1)	69 (77.5)	0.32
Chinese	199 (18.0)	4 (7.7)	181 (18.8)	14 (15.9)	
Indian	50 (4.5)	1 (1.9)	44 (4.6)	5 (5.6)	
Parity, *n* (%) 0	373 (33.8)	13 (24.5)	337 (35.0)	23 (25.8)	0.03
1	281 (25.4)	22 (41.5)	236 (24.5)	23 (25.8)	
>2	451 (40.8)	18 (34.0)	390 (40.5)	43 (48.3)	
Risk factors, *n* (%) Age above 35 Family history Previous GDM Obesity	328 (29.7) 248 (22.4) 189 (17.1) 157 (14.2)	12 (22.6) 6 (11.3) 11 (20.8) 10 (18.9)	281 (29.2) 222 (23.1) 150 (15.6) 126 (13.1)	35 (39.3) 20 (22.5) 28 (31.5) 21 (23.6)	0.07 0.14 0.001 0.02
Gestation in weeks at diagnosis, mean (SD)	24.6 (6.2)	23.1 (6.7)	24.6 (6.2)	24.7 (6.4)	0.22
Trimester of pregnancy at diagnosis, *n*(%) First	41 (3.7)	5 (9.4)	31 (3.2)	5 (5.6)	0.14
Second	593 (53.7)	29 (54.7)	519(54.0)	45 (50.6)	
Third	470 (42.5)	19 (35.8)	412 (42.8)	39 (43.8)	
Mean OGTT (mmol/L), mean (SD) Fasting Two hours	4.78 (0.64) 8.50 (0.83)	5.95 (0.34) 6.59 (0.94)	4.60 (0.47) 8.57 (0.68)	5.94 (0.31) 8.92 (0.81)	<0.001 <0.001
Infant Gestation at delivery (week)	38.3 (1.4)	38.2 (1.1)	38.3 (1.4)	37.8 (1.7)	0.004
Birthweight (kg)	3.09 (0.44)	3.17 (0.38)	3.08 (0.45)	3.15 (0.49)	0.09

**Table 2:** Pregnancy outcomes for the study population and the OGTT abnormalities subtypes.

Pregnancy Outcomes	Study Cohort * n = 1105	Subtypes of OGTT Abnormalities					
		*i*-Fasting * n = 53	OR (95% CI) AOR (95% CI) **	*i*-2hour * n = 963	OR (95%CI) AOR (95% CI) **	Both Abnormal * n = 89	OR (95%CI) AOR (95% CI) **
Insulin usage	96 (8.7)	11 (20.8)	2.98 (1.48–6.00) 2.94 (1.42–6.06)	62 (6.4)	0.22 (0.14–0.35) 0.23 (0.14–0.37)	23 (25.8)	4.50 (2.65–7.66) 4.14 (2.40–7.16)
Cesarean delivery	330 (29.9)	21 (39.6)	1.58 (0.90–2.78) 1.78 (0.98–3.21)	280 (29.1)	0.75 (0.52–1.09) 0.75 (0.51–1.10)	29 (32.6)	1.15 (0.72–1.83) 1.08 (0.67–1.76)
Preeclampsia	14 (1.3)	0 (0)	0.00 0.00	10 (1.0)	0.36 (0.11–1.17) 0.36 (0.11–1.21)	4 (4.5)	4.73 (1.45–15.41) 4.65 (1.38–15.70)
Stillbirth	5 (0.5)	1 (1.9)	5.04 (0.55–45.88) 3.65 (0.39–34.65)	4 (0.4)	0.59 (0.07–5.30) 0.63 (0.07–5.82)	0 (0)	0.00 0.00
Preterm delivery	75 (6.8)	2 (3.8)	0.53 (0.13–2.24) 0.54 (0.13–2.28)	63 (6.6)	0.76 (0.40–1.44) 0.79 (0.41–1.52)	10 (11.2)	1.84 (0.91–3.73) 1.72 (0.84–3.52)
Macrosomia	20 (1.8)	1 (1.9)	1.05 (0.14–7.96) 0.92 (0.12–7.21)	16 (1.7)	0.58 (0.19–1.77) 0.64 (0.21–2.00)	3 (3.4)	2.05 (0.59–7.13) 1.90 (0.53–6.80)
Shoulder dystocia	4 (0.4)	1 (1.9)	6.72 (0.69–65.76) 4.65 (0.44–49.16)	3 (0.3)	0.44 (0.05–4.27) 0.54 (0.05–5.41)	0 (0)	0.00 0.00
Neonatal hypoglycemia	7 (0.6)	1 (1.9)	0.00 0.00	4 (0.4)	0.19 (0.04–0.87) 0.21 (0.04–1.06)	3 (3.4)	8.78 (1.93–39.88) 6.95 (1.38–34.94)
Neonatal jaundice	77 (7.0)	1 (1.9)	0.25 (0.03–1.84) 0.28 (0.04–2.04)	70 (7.3)	1.51 (0.68–3.35) 1.30 (0.58–2.92)	6 (6.7)	0.96 (0.40–2.27) 1.12 (0.47–2.71)
NICU admission	38 (3.5)	1 (1.9)	0.00 0.00	32 (3.3)	0.78 (0.32–1.89) 0.82 (0.33–2.04)	6 (6.7)	2.21 (0.90–5.44) 2.11 (0.84–5.32)

i-Fasting, isolated fasting abnormality; i-2hour, isolated two-hour abnormality; OR, odds ratio; AOR, adjusted odds ratio; NICU, neonatal intensive care unit * Data presented as *n* (%) ** AOR: adjusted for maternal age, ethnicity, parity, and obesity.

**Table 3:** Comparison of clinical characteristics and pregnancy outcomes of gestational diabetes mellitus (GDM) women on insulin vs. diet therapy.

Parameter	Insulin (*n* = 96)	Diet Control (*n* = 968)	*p*-Value
Clinical Characteristics Age (years), mean (SD)	32.9 (4.5)	32.0 (4.4)	0.09
Gestation in weeks at diagnosis, mean (SD)	21.1 (5.7)	25.0 (6.0)	<0.001
Trimester of pregnancy at diagnosis First	9 (9.4)	25 (2.6)	<0.001
Second	67 (69.8)	506 (52.3)	
Third	20 (20.8)	436 (45.1)	
Mean OGTT (mmol/L), mean (SD) Fasting glucose	5.25 (0.74)	4.71 (0.60)	<0.001
2-h glucose	8.68 (1.00)	8.49 (0.81)	0.07
Risk factors, *n* (%) Age above 35	33 (34.4)	277 (28.6)	0.24
Family history	30 (31.3)	210 (21.7)	0.04
Previous GDM	29 (30.2)	151 (15.6)	0.001
Obesity	28 (29.2)	114 (11.8)	<0.001
Pregnancy Outcomes * Gestation at delivery (weeks), mean (SD)	37.4 (1.5)	38.4 (1.4)	<0.001
Birth weight (kg), mean (SD)	3.11 (0.56)	3.09 (0.43)	0.66
Cesarean delivery	43 (44.8)	270 (27.9)	AOR (95% CI) ** 2.03 (1.29–3.20)
Preeclampsia	5 (5.2)	9 (0.9)	6.11 (1.89–19.77)
Preterm birth	11 (11.7)	62 (6.4)	1.74 (0.86–3.50)
Stillbirth	2 (2.1)	2 (0.2)	12.64 (1.68–94.98)
Macrosomia	4 (4.2)	16 (1.7)	2.24 (0.71–7.10)
Neonatal hypoglycemia	4 (4.3)	2 (0.2)	18.48 (3.03–112.55)

SD, standard deviation; OGTT, oral glucose tolerance test; 2-h, two hour; AOR, adjusted odd ratio * Data presented as *n* (%) unless stated otherwise ** AOR: adjusted for maternal age, ethnicity, parity, and obesity.

**Table 4:** Associations between maternal risk factors and OGTT abnormalities.

OGTT Abnormalities	Maternal Age ≥ 35 AOR (95% CI) *p*-Value	Family history AOR (95% CI) *p*-Value	Previous GDM AOR (95% CI) *p*-Value	Obesity AOR (95% CI) *p*-Value
Isolated fasting	0.69 (0.33–1.45) 0.32	0.33 (0.14–0.80) 0.01	1.82 (0.85–3.89) 0.12	1.36 (0.66–2.82) 0.41
Isolated two hour	0.88 (0.58–1.35) 0.56	1.80 (1.11–2.91) 0.02	0.42 (0.26–0.67) <0.001	0.57 (0.36–0.89) 0.01
Both abnormal	1.44 (0.87–2.38) 0.16	0.22 (0.44–1.35) 0.77	2.42 (1.39–4.21) 0.002	1.90 (1.11–3.25) 0.02

OGTT, oral glucose tolerance test; AOR, adjusted odds ratio; CI, confidence interval; GDM, gestational diabetes mellitus. AOR adjusted for maternal age, ethnicity, parity, obesity, family history, and previous GDM.
